# Extrinsic Elastic Anisotropy in a Compositionally Heterogeneous Earth's Mantle

**DOI:** 10.1029/2018JB016482

**Published:** 2019-02-08

**Authors:** Manuele Faccenda, Ana M. G. Ferreira, Nicola Tisato, Carolina Lithgow‐Bertelloni, Lars Stixrude, Giorgio Pennacchioni

**Affiliations:** ^1^ Dipartimento di Geoscienze Università di Padova Padua Italy; ^2^ Department of Earth Sciences University College London London UK; ^3^ CERIS, Instituto Superior Tecnico Universidade de Lisboa Lisbon Portugal; ^4^ Department of Geological Sciences, Jackson School of Geosciences University of Texas Austin TX USA; ^5^ Department of Civil Engineering University of Toronto Toronto Ontario Canada

**Keywords:** seismic anisotropy, rock fabrics, compositional heterogeneities

## Abstract

Several theoretical studies indicate that a substantial fraction of the measured seismic anisotropy could be interpreted as extrinsic anisotropy associated with compositional layering in rocks, reducing the significance of strain‐induced intrinsic anisotropy. Here we quantify the potential contribution of grain‐scale and rock‐scale compositional anisotropy to the observations by (i) combining effective medium theories with realistic estimates of mineral isotropic elastic properties and (ii) measuring velocities of synthetic seismic waves propagating through modeled strain‐induced microstructures. It is shown that for typical mantle and oceanic crust subsolidus compositions, rock‐scale compositional layering does not generate any substantial extrinsic anisotropy (<1%) because of the limited contrast in isotropic elastic moduli among different rocks. Quasi‐laminated structures observed in subducting slabs using *P* and *S* wave scattering are often invoked as a source of extrinsic anisotropy, but our calculations show that they only generate minor seismic anisotropy (<0.1–0.2% of Vp and Vs radial anisotropy). More generally, rock‐scale compositional layering, when present, cannot be detected with seismic anisotropy studies but mainly with wave scattering. In contrast, when grain‐scale layering is present, significant extrinsic anisotropy could exist in vertically limited levels of the mantle such as in a mid‐ocean ridge basalt‐rich lower transition zone or in the uppermost lower mantle where foliated basalts and pyrolites display up to 2–3% Vp and 3–6% Vs radial anisotropy. Thus, seismic anisotropy observed around the 660‐km discontinuity could be possibly related to grain‐scale shape‐preferred orientation. Extrinsic anisotropy can form also in a compositionally homogeneous mantle, where velocity variations associated with major phase transitions can generate up to 1% of positive radial anisotropy.

## Key Points

- Rock‐scale layering in the Earth's mantle produces negligible extrinsic anisotropy
- Rock‐scale layering cannot be detected with seismic anisotropy but mainly with seismic wave scattering
- Grain‐scale layering can generate substantial extrinsic anisotropy around the transition zone

## 1 Introduction

Seismic anisotropy can result from the presence of a strain‐induced lattice preferred orientation (LPO) of minerals with anisotropic elastic properties, and/or of the shape‐preferred orientation (SPO) of isotropic compositional heterogeneities. The former anisotropy is referred to as intrinsic mechanical anisotropy, while the latter is referred to as extrinsic mechanical anisotropy. Extrinsic anisotropy occurs when (i) the size of the SPO is much smaller than the wavelength of the seismic signal and (ii) the contrast in isotropic elastic properties between the compositional domains is very large. When these conditions are satisfied, seismology fails to distinguish a finely layered and strongly heterogeneous isotropic medium from a smooth intrinsic anisotropic medium (Backus, 1962; Maupin & Park, 2015).

Within the Earth, extrinsic anisotropy can be related to either (1) the presence of a free gas or liquid phase included in elongated and preferentially oriented grain boundaries, pores, cracks, and porosity bands (Crampin, 1994; Holtzman & Kendall, 2010; Shapiro & Kaselow, 2005; Thomsen, 1995) or (2) grain‐scale (micrometer to centimeter) and/or rock‐scale (centimeter to kilometer) SPO of compositionally distinct domains. However, it is unclear whether the latter process could generate substantial extrinsic anisotropy.

Rock‐scale compositional anisotropy has been traditionally invoked to explain part of the seismic anisotropy measured within the lithospheric mantle (Bodin et al., 2015; Gee & Jordan, 1988; Kennett & Furumura, 2016). Indeed, there is seismological evidence for the presence of “fine” layering in the oceanic and continental lithosphere, such as 1‐ to 10‐km‐thick quasi‐laminated structures constrained from high‐frequency scattered waves (Furumura & Kennett, 2005; Garth & Rietbrock, 2017; Kennett & Furumura, 2008; Sun et al., 2014). In addition, grain‐scale and rock‐scale compositional anisotropy could explain the observed seismic anisotropy in regions, such as the mantle transition zone, where the intrinsic anisotropy of minerals is low and the presence of a free fluid phase is uncertain (Karato, 1998; Trampert & van Heijst, 2002). Globally, thermochemical mantle convection simulations suggest that compositional layering might be widespread within the Earth's interior (Ballmer et al., 2015; Olson et al., 1984a; van Keken et al., 2002). This is consistent with several seismic mantle tomography studies showing that rough 3‐D isotropic models can fit seismic data nearly as well as smoother 3‐D anisotropic models (e.g., Ferreira et al., 2010; Montagner & Jobert, 1988; Trampert & Woodhouse, 2003), although the differences in data fit become more substantial when very large and diverse data sets are used (Chang et al., 2014, 2015). Recent studies identified families of stable, fine‐scale models that are equivalent to long‐wavelength, vertical transversely isotropic (VTI) models (Alder et al., 2017; Bodin et al., 2015; Fichtner et al., 2013; Wang et al., 2013), and efforts to consider more general media are under way (e.g., Capdeville et al., 2010a, 2010b). However, it is unclear whether the equivalent fine‐scale models are compatible with the properties of Earth's mantle materials. For example, Wang et al. (2013) considered the 1‐D PREM model and demonstrated the existence of finely layered models compatible with PREM's lithospheric anisotropy. Yet, when analyzing their elastic properties, they found that the equivalent layered models demanded unrealistic contrasts in shear modulus of the two materials, incompatible with subsolidus petrological mantle models.

The aim of this study is to quantitatively estimate the strength of extrinsic anisotropy related to crustal and mantle compositional heterogeneities when sampled by different seismic phases. Although being an important source of seismic anisotropy, here we do not consider the presence of preferentially oriented, fluid‐filled cracks/pores/grain boundaries. By using *P*‐*T*‐dependent isotropic seismic properties and modal compositions of mafic and ultramafic rocks derived from thermodynamically consistent phase equilibria computed with HeFESTo (Stixrude & Lithgow‐Bertelloni, 2011), we show that extrinsic anisotropy at subsolidus, volatile‐free conditions might be relevant around the transition zone where strong contrasts in elastic moduli exist.

## 2 Structures Associated With Compositional Layering

### 2.1 Grain‐Scale SPO

**Figure 1:**
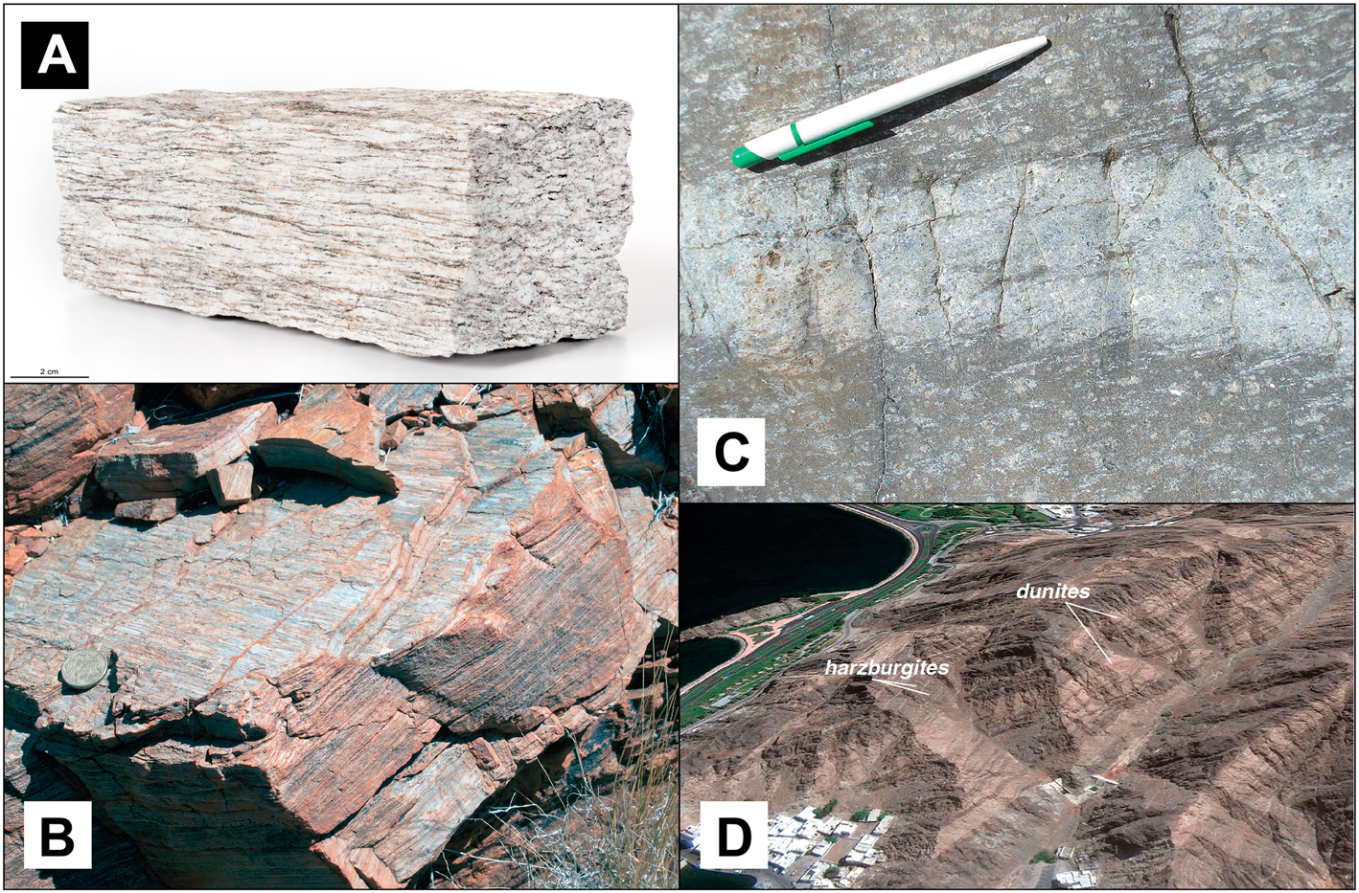
Compositional heterogeneities in exhumed rocks. (a) Gneiss from the Alps displaying elongated hard feldspar crystals surrounded by weak mica and quartz grains, yielding an irregular foliated pattern. (b) Granitoid from the Mushgrave complex, Australia, displaying planar foliation. (c) Centimeter‐scale pyroxenitic band embedded in spinel‐lherzolite in the Erro‐Tobbio unit, Voltri Massif, Ligurian Alps (Rampone & Borghini, 2008). Postmagmatic tectonic foliation is parallel to the pen and to the elongated pyroxene (white) crystals in the spinel‐lherzolite. (d) Decametric‐scale dunitic‐Harzburgitic layering in the crust–mantle transition zone of the Oman ophiolite (from Tommasi & Vauchez, 2015).

As a result of viscous deformation mineral aggregates and grains may progressively develop SPO elements of the rock fabric such as lineation (L) and foliation (S; Figures 1a–1c). Rocks dominated by a SPO of prolate (cigar‐shaped) and oblate (disk‐shaped) aggregates are referred to as L‐type and S‐type tectonite, respectively. The type of SPO associated with deformation of compositionally/rheologically distinct domains is the result of the imposed strain geometry and of the viscosity contrast between the rock‐forming mineral domains. Under noncoaxial deformation the SPO becomes progressively more strong and rotate into parallelism to the shear plane. This SPO may be destroyed (i) at low strain by postkinematic annealing, when the driving force becomes the reduction of the surface energy of the aggregate; (ii) at high strain during grain size sensitive creep (grain boundary sliding) due to phase mixing associated with dissolution/precipitation and nucleation processes (Kilian et al., 2011; Skemer et al., 2010; Tommasi & Vauchez, 2015); and (iii) by partial melting of the aggregate, when one of the secondary phases generating the compositional layering is removed (Almqvist et al., 2015). The latter mechanism is relevant, for instance, below mid‐oceanic ridges where peridotites can be homogenized by removal of pyroxene during reaction‐induced partial melting, yielding dunitic veins (Kelemen et al., 1995).

At large strains, a second‐order SPO may develop internally in recrystallized grains of monomineralic aggregates, oblique to the main foliation. This SPO results from the balance between elongation of grains in the direction on the instantaneous stretching axis (by either dilocation or diffusion creep) and rotation of grains due to the noncoaxial component of deformation. This type of SPO can be erased by postkinematic annealing.

A further form of SPO may arise from stabilization of elongated rigid grains (porphyroclasts) floating in a viscous flowing matrix. This may either occur in simple shear or in a general noncoaxial flow depending on particle‐matrix coherence, strain vorticity, and particle aspect ratio (Mancktelow et al., 2002; Passchier, 1987; Pennacchioni et al., 2001). This SPO only generally produces a limited anisotropy being related to a spatially nondense population of rigid porphyroclasts in the matrix. All the three types of above described grain‐scale crystal preferred orientation (CPO) may be simultaneously present within a mylonitic rock.

Few studies have attempted to measure extrinsic anisotropy related to grain‐scale compositional layering in crustal rocks. For example, (Kern et al., 2008) have estimated that in a strongly foliated biotitic gneiss about half of the measured Vp anisotropy (15%) is due to LPO of strongly anisotropic mica grains, while the remaining can be related to grain‐scale SPO (however, little or no Vs extrinsic anisotropy has been inferred in that case). Burlini and Kunze (2000) estimated a 2.6% Vp anisotropy in mylonitic Carrara marble where elongated calcite grains are interlayered with secondary white mica crystals, defining a foliation and lineation. It is important to note that grain‐scale SPO of secondary phases does not necessarily increase the total (intrinsic plus extrinsic) seismic anisotropy of a rock. For example, Tatham et al. (2008) pointed out that in the lower crust elongated ribbons of plagioclase dilute the intrinsic anisotropy of amphibole. Furthermore, seismic anisotropy is strongly diminished in crenulated rocks, where the early planar schistosity is overprinted by a later planar fabric (Naus‐Thijssen et al., 2011).

As crustal rocks, which are deformed by high‐temperature creep, display penetrative fabrics, it is logical to assume that similar structures could form in the hot mantle. Exhumed mantle shear zones exhibit monomineralic banding of olivine and pyroxene domains, although interlayered with fine‐grained polymineralic domains related to phase mixing processes where compositional layering has been erased (Linckens et al., 2014, 2011; Skemer et al., 2010; Tommasi & Vauchez, 2015). In general, however, mantle outcrops are part of the exhumed lithosphere, and hence they are not entirely representative of the hot mantle where a more diffused and long‐lasting deformation accommodated by high‐temperature creep takes place. Thus, it is instructive to numerically simulate the development of grain‐scale SPO in order to better understand which strain‐induced fabrics could potentially form in the hot sublithospheric mantle.

#### 2.1.1 Modeling Fabric Evolution in Two‐Phase Aggregates

The models reproduce the evolution of a two‐phase (matrix:inclusions = 70:30) system by Newtonian viscous flow and as a function of the amount of shear deformation and of the imposed viscosity contrast (numerical modeling details are reported in supporting information Text S1; Gerya, 2010). The inclusion and matrix isotropic viscosities are set to either 1 or 10, yielding three different cases with viscosity ratio (*η*_*c*_ = *η*_*i*_/*η*_*m*_) equal to 0.1, 1 and 10. We note that the models assume intracrystalline deformation and ignore grain size reduction by subgrain rotation recrystallization and grain‐boundary sliding processes that do not yield SPO. Furthermore, no recovery mechanism related to surface tension is considered, implying high capillary numbers. Hence, they represent upper bound estimates, although neglected pressure‐solution/precipitation processes and the presence of melt could accelerate further the shape change of grains (Zimmerman et al., 1999). The initial spherical shape of the inclusions is not representative of the crystal habit of several mantle phases.

**Figure 2:**
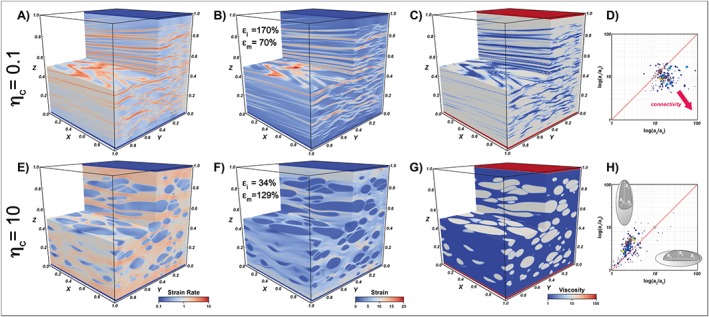
Three‐dimensional mechanical modeling of grain‐scale fabric development. Strain rate (panels a and e), cumulative strain (panels b and f), viscosity (panels c and g), and Flinn diagram (panels d and h) for a two‐phase aggregate with weak inclusions (panels a–d, *η*_c_ = 0.1) and hard inclusions (panels e–h, *η*_c_ = 10) at *γ* = 10. In the Flinn diagrams, the circle size and color is proportional to the inclusion volume, while the white dot is the bulk finite strain ellipsoid. Lateral merging of weak inclusions would increase the circle size shifting the position toward the bottom right corner of the diagram.

When the matrix and the inclusions have the same viscosity (*η*_*c*_ = 1), it is clear that the medium is mechanically homogeneous and plane strain (i.e., no deformation along the *Y* direction) establishes. The strain within the matrix and the inclusions is equal to the bulk strain (*ε*_*m*_ = *ε*_*i*_ = 100%), and the amount of bulk strain absorbed by each phase is equal to its volume fraction (*ε*_*bulk_m*_ = *ε*_*m*_ · *ϕ*_*m*_ = 70%, *ε*_*bulk_i*_ = *ε*_*i*_ · *ϕ*_*i*_ = 30%). The inclusions are perfectly aligned, and their shape is given by the bulk finite strain ellipsoid (white dot in Figures 2d and 2h), which in simple shear and plane strain plots over the diagonal of the Flinn diagram with coordinates $a_{1}/a_{2}=a_{2}/a_{3}=\sqrt{\exp\left(2\sinh^{-1}\left(\gamma /2\right)\right)}$.

When the inclusions are 10 times less viscous than the surrounding matrix (*η*_*c*_ = 0.1), the inclusions are flattened and tend to merge forming a foliated network (S‐type fabric) that absorbs a progressively higher amount of bulk deformation (Figures 2a–2c; *ε*_*bulk_i*_ = 55%, and *ε*_*i*_= 170% at *γ* = 10; Movie S1). The flat inclusions are not planar but wrap around elongated ribbons of the hard matrix. Conversely, when *η*_*c*_ = 10, the inclusions are deformed into constricted, elongated tubules that remain isolated and generate a lineated (L‐type) fabric (Figures 2e–2g; *ε*_*bulk_i*_  = 10%, *ε*_*i*_=34%), while the matrix accommodates most of the bulk deformation (*ε*_*bulk_m*_ = 90%, *ε*_*m*_= 129%; Movie S2). In the Flinn diagram, the weak and flattened inclusions plot mostly below the diagonal (Figure 2d), while the hard and constricted inclusions plot above it (Figure 2h).

Summarizing, *η*_*c*_~1 favors the formation of planar fabrics, while *η*_*c*_ < 1 the lateral connectivity of the weak inclusions at the expense of a more irregular layering. When inclusions are moderately stiffer than the matrix (*η*_*c*_ > 1), a lineated fabric establishes, while when *η*_*c*_ ≫ 1 (not shown here) the rigid inclusions preserve their original shape.

#### 2.1.2 Potential Strain‐Induced Grain‐Scale Fabrics in the Hot Oceanic Crust and Mantle

Although mafic and ultramafic rocks are polyphase aggregates formed by more than just two different types of minerals, for a wide range of *P*‐*T* conditions they can be approximated as two‐phase aggregates where the less abundant phase is surrounded by a more abundant matrix and other minor phases can be ignored. Thus, the model results presented in Text S2.1.1 can be used as a proxy of the typical grain‐scale fabrics found in the Earth's high‐temperature mantle.

In the upper mantle, olivine is the most abundant phase (60, ~75 and ~100% for a pyrolitic, harzburgitic and dunitic composition, respectively) and pyroxene (enstatite and, in fertile mantle, diopside) is less abundant (Stixrude & Lithgow‐Bertelloni, 2012). Pyroxene is known to be slightly harder than olivine, such that fabrics would be characterized by poorly flattened and elongated pyroxene grains.

With increasing depth, pyroxene is progressively absorbed by garnet, so that in the transition zone ultramafic rocks are made of a two‐phase aggregate with olivine polymorphs (wadsleyite or ringwoodite) and majoritic garnet (60:40 for a pyrolitic composition). In dry conditions, garnet appears to be harder than any other abundant mineral phases of the upper mantle and transition zone (Jin et al., 2001; Karato et al., 1995). Consequently, dry garnet grains should experience constrictional deformation or behave as a rigid inclusion, yielding lineated or no fabrics, respectively. In wet conditions, garnet becomes progressively weaker than olivine with increasing water content (Katayama and Karato, 2008), and at large strains the aggregate fabrics should be dominated by the flattened garnet crystals.

Decomposition of ringwoodite into bridgmanite and ferropericlase occurs at about 660‐km depth (postspinel reaction), while majorite progressively transforms to Ca‐ and Mg‐perovskite and disappears at about 720–750 km (postgarnet reaction; Hirose, 2002; Stixrude & Lithgow‐Bertelloni, 2011; see Faccenda & Dal Zilio, 2017, for a review). Below this depth, the uppermost lower mantle rocks can again be represented as a two‐phase aggregate of bridgmanite (which transforms to postperovskite below 2,650 km) and ferropericlase, with minor amounts Ca‐perovskite (70:30:0 and 76:17:7 phase proportions for a strongly depleted [dunitic] and fertile [pyrolitic] mantle compositions, respectively; Stixrude & Lithgow‐Bertelloni, 2012). Ferropericlase is estimated to be 3 orders of magnitude weaker than bridgmanite (Yamazaki & Karato, 2001) and accommodates most of the deformation, yielding a fabric with flattened ferropericlase crystals (Girard et al., 2016) that at large strains could be analogous to that shown in Figures 2e–2g. However, in pyrolites the volume fraction of ferropericlase is almost half that present in the samples deformed by Girard et al. (2016), such that it is unclear whether full interconnectivity of periclase crystals and development of penetrative foliation would take place at such low concentrations.

In the oceanic crust, mafic rocks transform to eclogites in the 30‐ to 60‐km depth range at equilibrium. Eclogites are made of omphacitic pyroxene and pyrope garnet, plus less abundant quartz (10%). In dry eclogites, omphacite accommodates most of the deformation and garnet crystals behave essentially as rigid inclusions; in wet eclogite, the deformation is accommodated by shape change in both garnet and omphacite, yielding quasi‐laminated fabrics (Zhang & Green, 2007). Pyroxene is progressively absorbed by garnet, so that in the transition zone mafic rocks are garnetitic plus 10% of stishovite. When stishovite is weaker than garnet, a foliated (but poorly interconnected due to its low volume fraction) fabric can establish. In the lower mantle, the subducted crust is formed by a four‐phase aggregate with about similar volume fractions and unknown relative strength, such that it is not yet possible to predict potential grain‐scale fabrics.

### 2.2 Rock‐Scale SPO

Magmatic differentiation is the principal mechanism generating rock‐scale heterogeneities. The magmatically differentiated oceanic lithosphere is often represented by an enriched basaltic crust overlying a depleted harzburgitic mantle and a deeper lherzolitic phase, which is the most fertile mantle. However, exposed mantle sections indicate that the lithosphere is heterogeneous at the centimeter to meter scale. For example, oceanic peridotites from the Oman and the Ingalls Ophiolites are characterized by the interlayering of lherzolitic and harzburgitic levels, together with the presence of 5% to 15% of dunitic layers, up to 5% of pyroxenitic veins, and elongated gabbroic lenses near the Moho (Hirschmann & Stopler, 1996; Jousselin et al., 2012; Kelemen et al., 2000, 1995; Figure 1d). The different compositional domains can be tabular, lensoid, or cylindrical in shape (Kelemen et al., 2000). The continental lithospheric mantle appears to be more compositionally heterogeneous, displaying a larger volume fraction of pyroxenites and peridotitic levels impregnated with plagioclase near the Moho (Downes, 2007; Rampone & Borghini, 2008; Figure 1c).

The orientation of the compositional domains, which depends on magmatic differentiation and, more importantly, deformation processes, is typically parallel to the high‐temperature foliation. In the oceanic mantle lithosphere heterogeneities may become parallel and thinned by shearing associated with corner flow at spreading ridges (Braun & Kelemen, 2002). For example, dunites from the Oman Ophiolite are mostly concordant with harzburgites and parallel to foliation and to the Moho (Kelemen et al., 1995). Synkinematic reactive melt percolations, leading to melt segregation in layers subparallel to the shear plane, have also been proposed as a mechanism producing anastomosed to planar gabbro layering in plagioclase lherzolites of the Lanzo ophiolite (Higgie & Tommasi, 2014) and the pyroxenitic layering in the Oman ophiolite Moho transition zone (Jousselin et al., 2012; Soustelle et al., 2014). The layering and the high‐temperature foliation are sometimes crosscut by highly discordant dunitic and pyroxenitic dikes that are generally undeformed and form off‐axis in residual peridotites (Boudier & Coleman, 1981; Kelemen et al., 1995). These quasi‐laminated structures appear to be constrained at depth by high‐frequency scattered *P* waves (e.g., Furumura & Kennett, 2005; Sun et al., 2014).

Upon subduction, the compositionally stratified lithosphere is recycled back in the convective hot mantle and experiences progressive conductive warming and viscosity reduction. The weakened chemically distinct heterogeneities are stirred into the surrounding mantle matrix by convective laminar or turbulent shear flow, and the distance between layers progressively decreases with deformation (Olson et al., 1984b). As a result, a marble‐cake mantle model containing elongated strips of subducted oceanic lithosphere that have been stretched and thinned by deformation associated with mantle convection has been proposed by Allegre and Turcotte (1986) and reproduced with 2‐D numerical simulations (e.g., Ballmer et al., 2015; Nakagawa et al., 2010; van Keken et al., 2002). In the creeping mantle, layering becomes parallel to the foliation or shear plane for sufficiently high strains or maximum axis of the finite strain ellipsoid (FSE) (Olson et al., 1984b), although complex mixing patterns may arise in 3‐D simulations due to turbulent flow (Ferrachat & Ricard, 1998).

Layering is destroyed by several mechanisms, such as (i) dissolution processes, including recrystallization and solid‐state diffusion; the latter process is efficient for distances of few centimeters over hundreds of million years (Allegre & Turcotte, 1986; Stixrude & Lithgow‐Bertelloni, 2012); (ii) necking of rigid layers into ribbon‐like boudins (Schmalholz & Maeder, 2012); (iii) reprocessing of the mantle at an oceanic ridge (Allegre & Turcotte, 1986); and (iv) mechanical unmixing related to phase transformations and density contrasts among different compositional heterogeneities (Ballmer et al., 2015; Faccenda & Dal Zilio, 2017).

## 3 Quantification of Extrinsic Seismic Anisotropy

In this section, we quantify extrinsic anisotropy in layered (Text S3.1) and nonlayered (Text S3.2) media by using effective medium theories and isotropic elastic moduli of mafic and ultramafic rocks. These estimates are representative of situations where seismic waves propagate parallel to fabrics characterized by a strong SPO (perfect layering or, in nonlayered media, perfect alignment of the inclusions). In Text S3.3 we show the dependence of extrinsic anisotropy on the seismic wave incidence angle for these strong fabrics. Finally, extrinsic anisotropy resulting from weaker SPO fabrics is estimated by simulating the propagation of 3‐D seismic wavefronts through the modeled strain‐induced microstructures shown in Figure 2 and Movies S1 and S2 (Text S3.4).

Seismic anisotropy is quantified in terms of radial and azimuthal anisotropy for both *S* waves (*R*_*S*_, *A*_*S*_) and *P* waves (*R*_*P*_, *A*_*P*_) defined as

(1) $$\begin{array}{c} R_{S}=N/L-1=V_{\mathrm{SH}}^{2}/V_{\mathrm{SV}}^{2}-1\\ R_{P}=\frac{A}{C}-1=\frac{V_{\mathrm{PH}}^{2}}{V_{\mathrm{PV}}^{2}}-1\\ A_{S}=C_{55}/C_{44}-1\\ A_{P}=C_{11}/C_{22}-1 \end{array}$$

where *V*_*SH*_ and *V*_*SV*_ are the azimuthally averaged horizontally and vertically polarized *S* waves speeds; *V*_*PH*_ and *V*_*PV*_ the azimuthally averaged horizontally and vertically propagating *P* waves speeds; while *A*, *C*, *L*, and *N* are the Love elastic constants defined in Text S2 (e.g., Montagner & Nataf, 1986). Horizontal layering yields maximum *R*_*S*_ = *C*_66_/*C*_44_ − 1, *R*_*P*_ = *C*_11_/*C*_33_ − 1, which is equivalent to the maximum *A*_*S*_ = *C*_66_/*C*_44_ − 1, *A*_*P*_ = *C*_11_/*C*_33_ − 1 occurring for vertical layering.

Another useful measure of seismic anisotropy is the *S* wave birefringence which is defined here as:

(2) $$\mathrm{AVs}=\left({\mathrm{Vs}}_{1}-{\mathrm{Vs}}_{2}\right)/{\mathrm{Vs}}_{2}$$

where *Vs*_1_ and *Vs*_2_ are the fast and slow *S* waves along the propagation direction calculated by solving the Christoffel equation. When the layering is horizontal, ${\mathrm{AV}}_{S}=\sqrt{R_{S}+1}-1$.

*AVs* is typically used to estimate the polarization anisotropy of body *S* waves along the direction of propagation (e.g., SKS splitting), while *R*_*S*_ and *A*_*S*_ are used to measure the (squared) velocity variation of horizontally traveling *S* waves (e.g., surface waves) with the direction of polarization and propagation, respectively.

### 3.1 Layered Media

We compute the elastic tensors of perfectly layered media displaying grain‐scale and rock‐sale SPO using the Smooth Transversely Isotropic Long‐Wavelength Equivalent effective medium theory (Backus, 1962; Text S3.1). We assume that the layering is horizontal (VTI medium), which yields maximum radial anisotropy and null azimuthal anisotropy. Thus, the *R*_*S*_ and *R*_*P*_ estimates that we obtain must be considered upper bounds. Maximum *A*_*S*_ and *A*_*P*_ are for vertical layering (HTI medium), with magnitudes that are equivalent to the maximum *R*_*S*_ and *R*_*P*_ (for VTI medium) discussed below.

**Figure 3:**
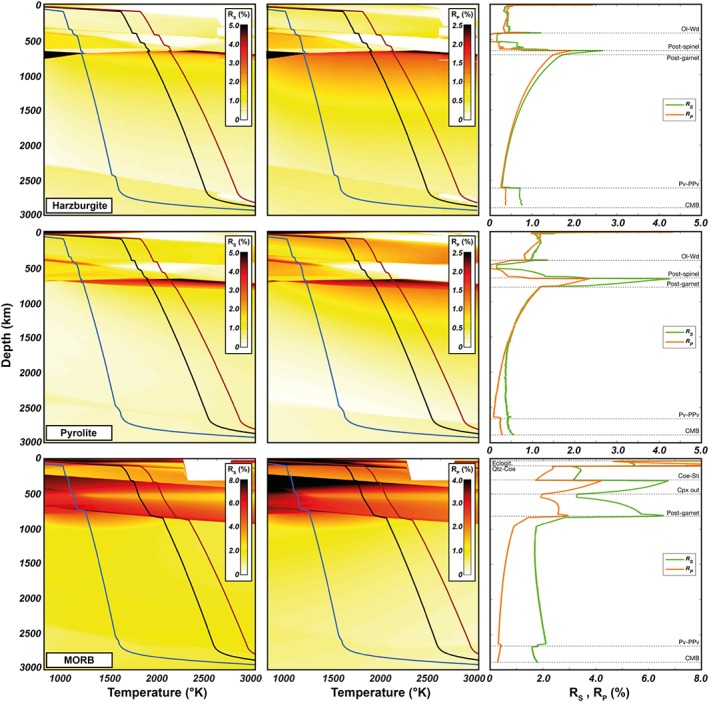
Radial anisotropy due to grain‐scale shape‐preferred orientation for pyrolitic, harzburgitic, and MORB compositions. Dunites are ignored because of the absence of abundant secondary phases. *R*_S_ (left column) and *R*_P_ (middle column) have been computed for a perfectly layered Smooth Transversely Isotropic Long‐Wavelength Equivalent using the mineral isotropic elastic constants and volume fractions for a range of mantle *P*‐*T* conditions. The blue, black, and red curves are the 1000‐, 1600‐, and 1800‐K isentropes (100–2,600 km) with an upper boundary layer with surface temperature of 273 K and a lower boundary layer that reaches a temperature of 4100 K (Nimmo et al., 2004; Stixrude et al., 2009). (right column) The green and orange lines are *R*_s_ and *R*_P_ along the 1600‐K isentrope. The dashed horizontal lines indicate major phase transitions. Ol = Olivine; Wd = Wadsleyite; Pv = Perovskite; PPv = Post‐Perovskite; Coe = Coesite; Sti = Stishovite; Cpx = Clinopyroxene; CMB =Core‐Mantle Boundary;MORB = mid‐ocean ridge basalt.

Radial anisotropy associated with grain‐scale layering (i.e., perfect foliation) in pyrolite, harzburgite, and mid‐ocean ridge basalt (MORB) is shown in Figure 3. Average elastic moduli are computed by using as weights the volume fractions of mineral phases taken from phase equilibria calculations. In pyrolite, *R*_*S*_ and *R*_*P*_ are ≤1%, except in between the postspinel and the postgarnet phase transitions where *R*_*S*_ ~ 2–4% and *R*_*P*_ ~ 1–2% due to the delayed transformation of garnet into bridgmanite (Hirose, 2002; Stixrude & Lithgow‐Bertelloni, 2012). In harzburgite, extrinsic anisotropy is smaller than that in pyrolite because of its more depleted composition and higher abundance of the olivine component at the expense of the pyroxene‐garnet component. Perfectly foliated basalts display the highest extrinsic anisotropy, with *R*_*S*_ that is 3–6% down to the postgarnet phase transitions (about 825 km) and about 2% below it, while *R*_*P*_ is 2–4% (with peaks of 8%) above the postgarnet phase transition and < 1% in the rest of the lower mantle.

**Figure 4:**
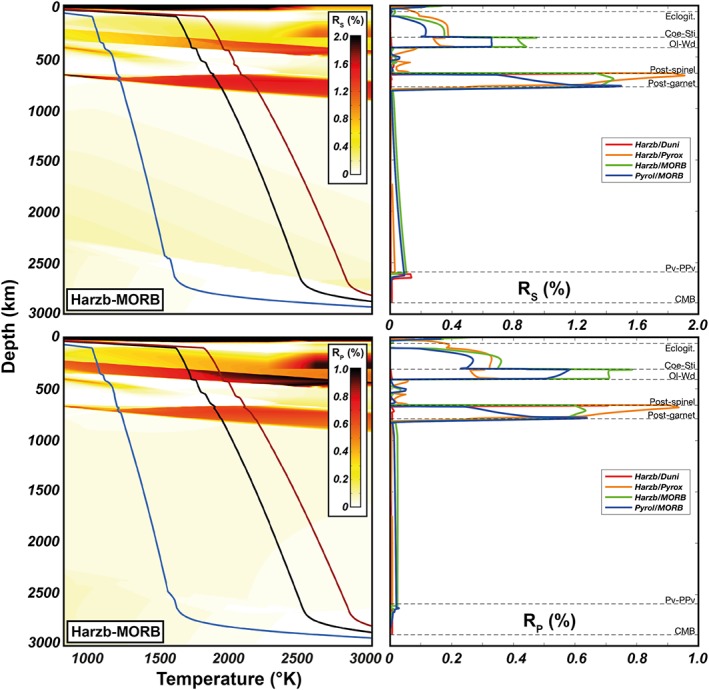
Radial anisotropy due to rock‐scale SPO for different periodic isotropic two‐layered (PITL) rock mixtures. *R*_S_ and *R*_P_ have been computed for 50:50 mixtures, which yields the maximum anisotropy (Text S3.1). *R*_S_ (top row) and *R*_P_ (bottom row) are shown for a range of mantle *P*‐*T* conditions for the MORB:Harz mixture (left column; the blue, black, and red curves are the 1000‐, 1600‐, and 1800‐K isentropes) and along the 1600‐K adiabat for different PITL mixtures (right column). Ol = Olivine; Wd = Wadsleyite; Pv = Perovskite; PPv = Post‐Perovskite; Coe = Coesite; Sti = Stishovite; Cpx = Clinopyroxene; CMB =Core‐Mantle Boundary; MORB = mid‐ocean ridge basalt.

Radial anisotropy related to rock‐scale SPO in two‐phase mixtures with equal volume fraction of each component (yielding maximum anisotropy) is shown in Figure 4. The anisotropy is highest for mixtures composed of both mafic and ultramafic rocks and minor for mixtures of either mafic or ultramafic (e.g., dunite mixed with harzburgite) rocks. In the former case, the anisotropy is significant only in between the coesite‐stishovite/olivine‐wadsleyite (*R*_*S*_ and *R*_*P*_ = 0.5–1%) and postspinel/postgarnet (*R*_*S*_ = 1–2%, *R*_*P*_= 0.5–1%) phase transitions. In the unlikely situation where the entire mantle section is subvertically layered and composed at any depth by a Harz:MORB = 50:50 mixture, an *S* wave traveling from the base to the top of the mantle, such as core‐refracted SKS phases, would accumulate a maximum 0.4 s of splitting delay time.

**Figure 5:**
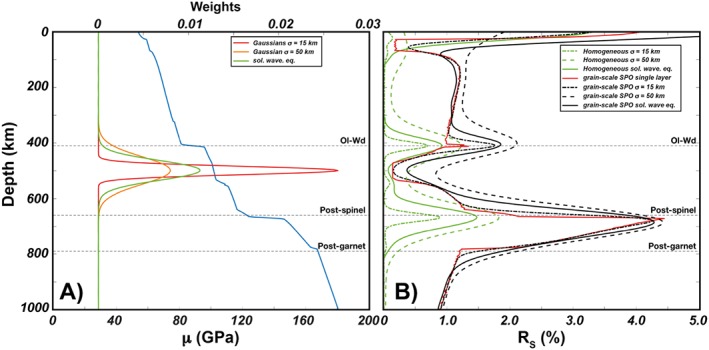
Radial anisotropy as measured by long‐period, horizontally propagating shear waves. (a) Examples of weight functions peaking at 500‐km depth. The blue curve is the shear modulus along the reference profile. (b) Radial anisotropy for a homogeneous isotropic pyrolite and due to grain‐scale SPO for a pyrolite. We use gaussian curves (dashed and dot‐dashed lines) as well as more realistic normal mode solutions of the wave equation (Capdeville et al., 2013; solid lines) as weight functions for the Smooth Transversely Isotropic Long‐Wavelength Equivalent to estimate the polarization anisotropy. The red curve is radial anisotropy due to grain‐scale layering present at a given depth (as in Figure 3). SPO = shape‐preferred orientation; Ol = Olivine; Wd = Wadsleyite; Pv = Perovskite; PPv = Post‐Perovskite; Coe = Coesite; Sti = Stishovite; Cpx = Clinopyroxene; CMB = Core‐Mantle Boundary.

Long‐period body and surface waves are sensitive to a range of depth intervals rather than to a single layer as it was assumed so far. Hence, we have used gaussian curves as well as more realistic normal mode solutions of the wave equation (Capdeville et al., 2013) as weight functions for the Smooth Transversely Isotropic Long‐Wavelength Equivalent to estimate the polarization anisotropy along the reference pyrolytic mantle profile displaying a perfectly layered grain‐scale SPO (see Text S4; Capdeville et al., 2013; Ferreira et al., 2010). Thus, the elastic moduli of mineral phases are weighted by their volume fraction at a given depth and as a function of the depth‐dependent sensitivity of the gaussian curves/normal mode solutions of the wave equation. We have also repeated the same exercise for a homogeneous pyrolitic mantle composition to isolate the contribution of compositional heterogeneities from that due to the increase with depth of the elastic moduli associated with phase transitions and the increase of pressure. The radial anisotropy is generally higher than for the single layer case because a larger range of elastic moduli are sampled (Figure 5). On the other hand, it is interesting to observe that major phase transitions can account for up to about half of the observed signal sampled at those depths. Hence, up to 1% of positive radial anisotropy measured around the transition zone might not necessarily indicate the presence of horizontally elongated compositional heterogeneities.

**Figure 6:**
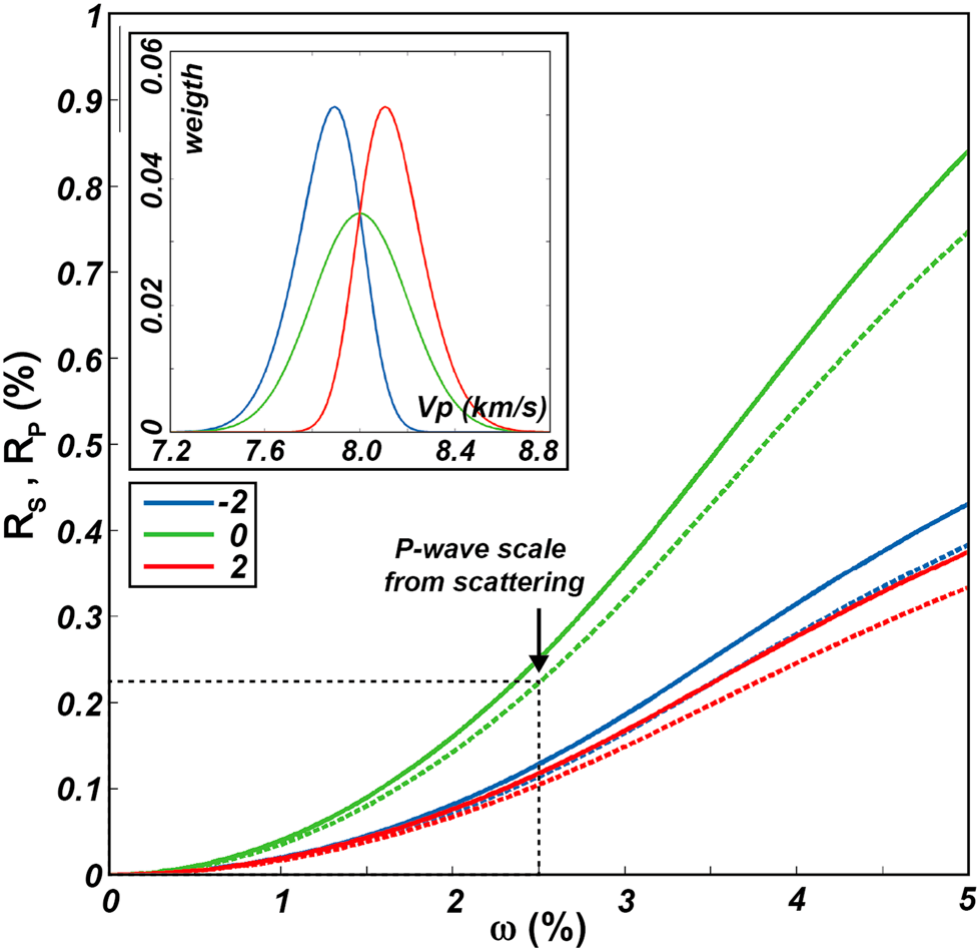
Radial anisotropy in multicomponent systems. *R*_S_: continuous lines, *R*_P_: dashed lines. Symmetric and asymmetric distributions are given by $w\left(x\right)=\frac{2}{\omega }\phi \left(\frac{x-\xi }{\omega }\right)\Phi \left(\alpha \left(\frac{x-\xi }{\omega }\right)\right)$, where $\phi \left(x\right)=\frac{1}{\sqrt{2\pi }}e^{-\frac{x^{2}}{2}}$, $\Phi \left(x\right)=\frac{1}{2}\left[1+\mathrm{erf}\left(\frac{x}{\sqrt{2}}\right)\right]$, *ξ* is the location (Vs = 4,619 m/s; Vp= 8,000 m/s; Poisson ratio = 0.25; density = 3,300 kg/m^3^), *ω* is the scale (for symmetric—Gaussian—distributions it is the standard deviation; shown as percentage of *ξ*), *α* is the shape (−2, 0, 2 for left‐skewed, symmetric, right‐skewed distributions). Inset: Vp distributions with *ω* = 2.5% (as from *P* wave scattering data in the lithosphere; e.g., Furumura & Kennett, 2005; Sun et al., 2014), which generate radial anisotropy of about 0.1–0.2%. In a two‐component system with *ω* = 2.5% and 50:50 phase proportions, *w*_Vs_ (4,537 m/s, 4,700 m/s) = *w*_Vp_ (7,859 m/s, 8,141 m/s) = (0.5, 0.5), and radial anisotropy is about 0.1 (%).

A better way to represent multicomponent systems such as the lithosphere in proximity of the Moho transition zone (Higgie & Tommasi, 2014; Jousselin et al., 2012) is by modeling the range of seismic velocities with skew‐normal distributions. Obviously, the wider the seismic velocity distribution, the higher is the radial anisotropy (Figure 6). For skew‐normal distributions analogous to those inferred from the scattering of *P* waves propagating through the lithosphere (Furumura & Kennett, 2005; Sun et al., 2014), radial anisotropy is only about 0.2% when the distribution is symmetric, and progressively decreases with increasing asymmetry of the distribution.

### 3.2 Nonlayered Media

The elastic properties of two‐phase aggregates composed by inclusions dispersed in a matrix are modeled with the Differential Effective Medium, which, among other effective medium methods, better estimates the medium elastic properties at high inclusion concentrations (Text S3.2; Hornby et al., 1994; Mainprice, 2007; McLaughlin, 1977). The shapes of the isolated inclusions range from uniaxial prolate ellipsoids (*a*_1_ : *a*_2_ : *a*_3_ = 50:1:1; representative of a L‐type fabric) to oblate ellipsoids (50:50:1; yielding foliation). It is important to notice that the calculated extrinsic anisotropy is again an upper bound estimate, as the inclusions are assumed to be perfectly oriented in the same direction, yielding maximum SPO.

**Figure 7:**
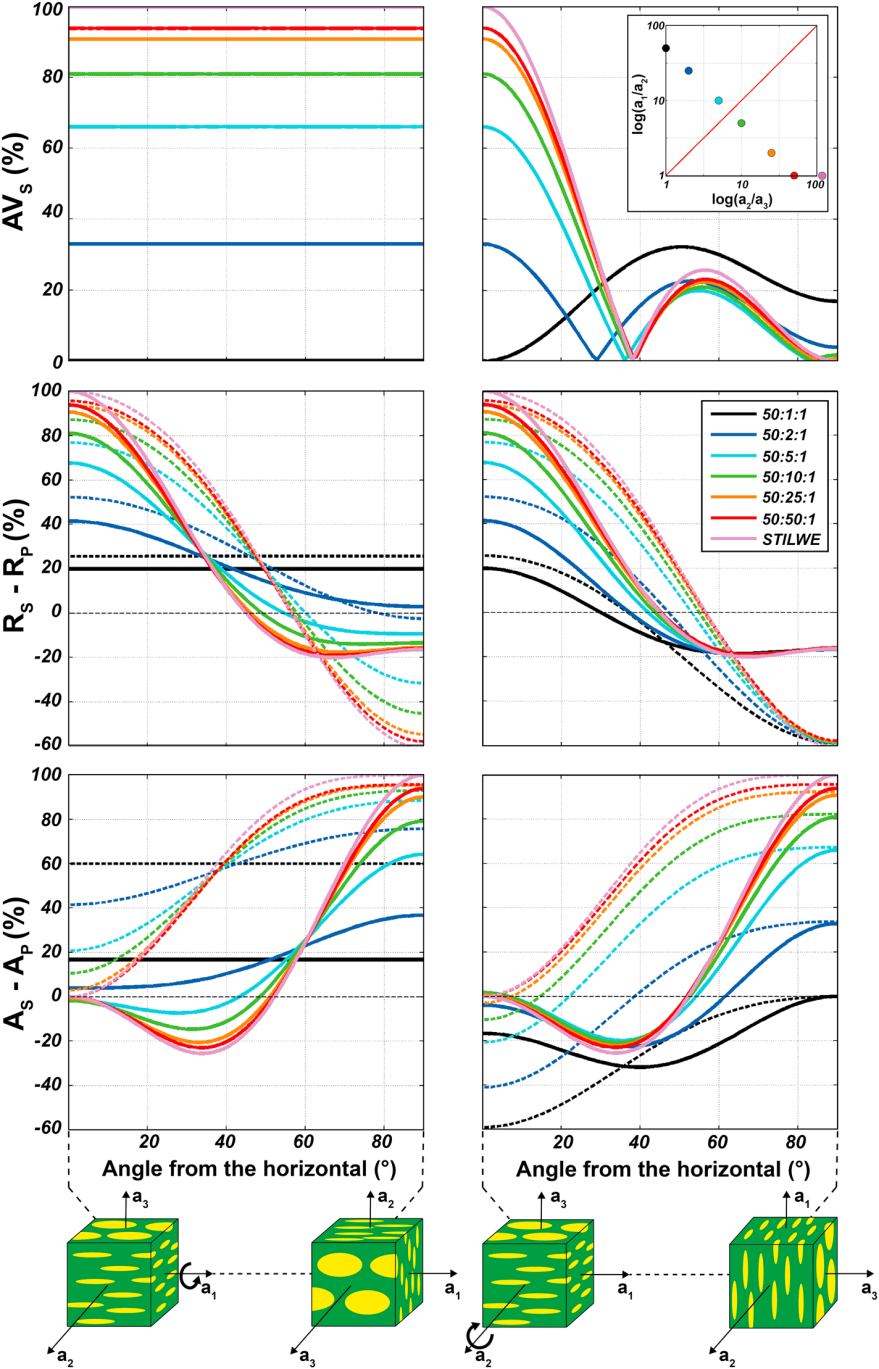
Seismic birefringence and radial and azimuthal anisotropy as a function of the heterogeneity shape and wave incidence angle. Continuous and dashed lines indicate, respectively, *S* wave and *P* wave anisotropies. Different colors correspond to different inclusion aspect ratios. The two‐phase medium is rotated along the longest (a_1_, left column) and intermediate (a_2_, right column) axes of the inclusions. AV_S_ (top row), *R*_S_, *R*_P_ (middle row), and *A*_S_, *A*_P_ (bottom row) are normalized to the maximum value obtained for a layered STILWE medium (purple lines). AV_S_ is calculated for *S* waves propagating along the direction parallel to the a_1_ axis when horizontal. Negative azimuthal anisotropy indicates that the fast seismic wave is normal to the rotation axis. The inset in the top right panel is a Flinn diagram where the different inclusion shapes are plotted. STILWE = Smooth Transversely Isotropic Long‐Wavelength Equivalent.

The shear wave that is polarized parallel to the flat inclusions travels faster than the shear wave polarized perpendicular to the faces for both fast and slow inclusions. Similarly to (Kendall, 2000) we found that extrinsic anisotropy is low for prolate (cylindrical) secondary phases and increases with the degree of flattening of the inclusions toward the theoretical upper bound associated with layered media (Figure 7).

### 3.3 Extrinsic Anisotropy as a Function of the Incidence Angle

The dependency of extrinsic anisotropy on the incidence angle has been estimated by rotating the elastic tensors around the fabric structural axes (Figure 7). For a layered or foliated (disc‐shape inclusions) medium, *AVs* is highest when shear waves propagate subparallel to the layers or to the inclusion flat faces, and rapidly diminishes with increasing incidence angle such that it is null when perpendicular to them (Figure 7, top row). *R*_*S*_ and *R*_*P*_ are maximum and positive when the layering is horizontal, 0 when ~ 45°, and negative for higher incidence angles, although with magnitudes that are 20% and 60% of the horizontal case for *S* and *P* waves, respectively (Figure 7, middle row). In contrast, *A*_*S*_ is 0 for horizontal layering, negative between 0 and about 50°, and then positive with a peak of the anisotropy for vertical layering. *A*_*P*_ is small or 0 for horizontal foliation and maximum for vertical foliation (Figure 7, bottom row).

For a lineated medium, radial anisotropy is positive or negative when the cylindrical inclusions are either horizontal or vertical, respectively. *A*_*S*_ displays a peak for rods inclined at 30° and 50° from the horizontal direction and decreases to 0 when the rods are vertically oriented. *A*_*P*_ is maximum for horizontal rods and upon their rotation it decreases progressively to 0.

These results are valid when the inclusions are either faster or slower than the matrix.

### 3.4 Irregularly Lineated or Foliated Rocks

In nature the strength of the SPO is typically weaker than in our calculations as secondary mineral/rock phases and their boundaries are unlikely to be perfectly aligned and planar as assumed so far but are arranged in irregular lineated or foliated patterns roughly analogous to those shown in Figure 2. No analytical solution exists to estimate the elastic anisotropy of such media. Thus, we performed 3‐D seismic wave propagation experiments with the software Sofi3D (Text S5; Bohlen, 2002) in a perfectly layered medium and in the foliated and lineated models shown in Figure 2, to estimate the shear wave velocities along different propagation and polarization directions (Table 1 and Figure S4). Input models (236 × 236 × 236 nodes) of compressional wave velocity, shear wave velocity, and density were directly obtained from the deformation experiments. To simulate the propagation of shear waves in such models, we applied shear displacement on three orthogonal sides of each model (layered, foliated, and lineated) and recorded the three components of the particle velocity on the opposite side. As a consequence, for each model, we simulated propagation along three orthogonal directions. To evaluate anisotropy of shear waves, we compared the propagation speed of two orthogonally polarized shear waves for each direction of propagation. Thus, we performed a total of 18 experiments. In each experiment, uncertainties on shear wave velocities and shear wave anisotropy were <0.5% and <0.011%, respectively.

**Table 1:** Results From 3‐D Seismic Wave Propagation Experiments

Model	Vs_*XY*_	Vs_*XZ*_	AVs_*X*_	AVs_X_*	Vs_*YX*_	Vs_*YZ*_	AVs_*Y*_	AVs_Y_*	Vs_*ZX*_	Vs_*ZY*_	AVs_*Z*_	AVs_Z_*
Layered	4,049.4	3,989	1.51	100	4,049.4	3,989.2	1.51	100	3,991.9	3,991.9	0	0
Foliated	4,172.6	4,126	1.13	74.83	4,140.4	4,084.5	1.37	90.73	4,042.5	4,041.1	0.03	1.99
Lineated	4,133.1	4,104	0.7	46.36	4,111.5	4,076	0.87	57.62	4,049.4	4,053.6	0.1	6.62

*Note*. Shear wave velocities are in meters per second (the first index indicates the propagation direction *i*, the second the direction of particle oscillation; *X* is the shearing direction, *Z* the normal to the shear plane), AVs_*i*_ = (Vs1 − Vs2)/Vs2 * 100, AVs_i_* = AVs_*i*_/AVs_MAX_ * 100, where AVs_MAX_ occurs when shear waves propagate in the layered medium parallel to the layering. In the layered medium, the volume fraction of the fast layers is 28%, which explains the lower absolute velocities when compared to the foliated and lineated models where the volume fraction of the faster phase is 30%. Fast phase: *K* = 120 GPa, *μ* = 72 GPa. Slow phase: *K* = 80 GPa, *μ* = 48 GPa.

As expected, shear wave extrinsic anisotropy is strongest when waves propagate parallel to perfectly planar layers. On the other hand, the anisotropy is null or negligible when shear waves propagate normal to the shear plane. For shear waves propagating in the shear plane, the anisotropy is still 75–90% of the layered case in strongly foliated media and 46–58% in media with elongated fast inclusions. This agrees reasonably well with the anisotropy predicted in Figure 7 for regularly oriented inclusions with shapes compatible to those reported in the Flinn diagrams in Figures 2d and 2h. Thus, although irregular, a well‐developed fabric can still produce substantial extrinsic anisotropy when compared to the theoretical maximum, despite being significantly reduced.

## 4 Discussion

Quantification of extrinsic anisotropy indicates that grain‐scale SPO is potentially more important than rock‐scale layering, although the contrast in elastic moduli and the SPO strength are generally not sufficient to generate significant extrinsic anisotropy in most regions of the Earth's mantle. At the rock scale, layering of an ideal and probably unrealistic mixture (same volume fractions) of harzburgite and MORB can produce up to 0.5–1% and 1–2% of radial anisotropy right above and below the transition zone, respectively. In a perfectly foliated pyrolite, grain‐scale SPO could be relevant in the uppermost lower mantle where the coexistence of majoritic garnet with bridgmanite results in *R*_*S*_= 2–4% and *R*_*P*_= 2%. In perfectly foliated basalts, instead, *R*_*S*_ is large throughout the mantle, especially at shallow depths (<100 km) and in between 300‐ and 825‐km depth where also *R*_*P*_ is quite significant.

These upper bound estimates are sensibly reduced in irregularly foliated/layered or nonlayered media, such that extrinsic anisotropy is minor when the solid heterogeneities are cylindrical and/or in the presence of a weak SPO. To which degree then are compositional heterogeneities laterally continuous and perfectly aligned within the convecting mantle?

At the rock‐scale, the oceanic crust formed at intermediate‐ to fast‐spreading ridges certainly constitutes a distinct and laterally continuous layer in the oceanic lithosphere, while that formed at slow‐spreading ridges is rather irregular. Within the lithospheric mantle, geological data indicate that grain‐scale and rock‐scale compositional domains are not perfectly layered but can form anastomosed and lensoid patterns (Kelemen et al., 2000; Tommasi & Vauchez, 2015). These irregular patterns would decrease the amount of extrinsic anisotropy by at least 10–30% (Table 1). On the one hand, the irregular layering could eventually become more planar and parallel by convective shear deformation. On the other hand, however, rheological contrasts among different rocktypes could lead to disruption of the layering. For example, a less abundant but stiffer layer such as the garnet‐rich (300‐ to 825‐km depth) oceanic crust embedded in a softer mantle would be progressively stretched leading to pinch‐and‐swell structures that will decrease the amount of extrinsic anisotropy.

At the grain scale, our mechanical simulations suggest that mineral aggregates composed by weak secondary phases develop irregular but interconnected foliation. Thus, a foliated uppermost lower mantle (weak ferropericlase inclusions) could effectively generate significant extrinsic anisotropy given the large contrast in isotropic elastic moduli of the coexisting mineral phases. Presence of foliated fabrics in the garnet‐rich oceanic crust (forming when stishovite acts a weak inclusion) can locally produce high seismic anisotropy, provided the basalt volumetric fraction is high. Mantle convection simulations have demonstrated that density contrasts among the different rock components may favor mechanical unmixing and accumulation of MORB material in the transition zone (40–50%) as well as at the base of the lower mantle (e.g., Ballmer et al., 2015). Hence, extrinsic anisotropy related to grain‐scale SPO in MORB material may explain the *S* and *P* wave azimuthal anisotropy measured in the intrinsically isotropic lower transition zone (Chang et al., 2015; Trampert and van Heijst, 2002; Wei et al., 2015; Yuan & Beghein, 2013). It is worth to mention that SiO_2_ polymorphs have large intrinsic anisotropy (Karki et al., 1997), and Cordier et al. (2004) showed that stishovite aggregates develop a LPO at transition zone depths with 8.9% and 7.1% of *P* and *S* wave anisotropies. However, in the lower transition zone with an average 40–50% basalt content, the amount of stishovite would be about 4–5% and the amount of seismic anisotropy related to the stishovite LPO would be less than 0.5%.

**Figure 8:**
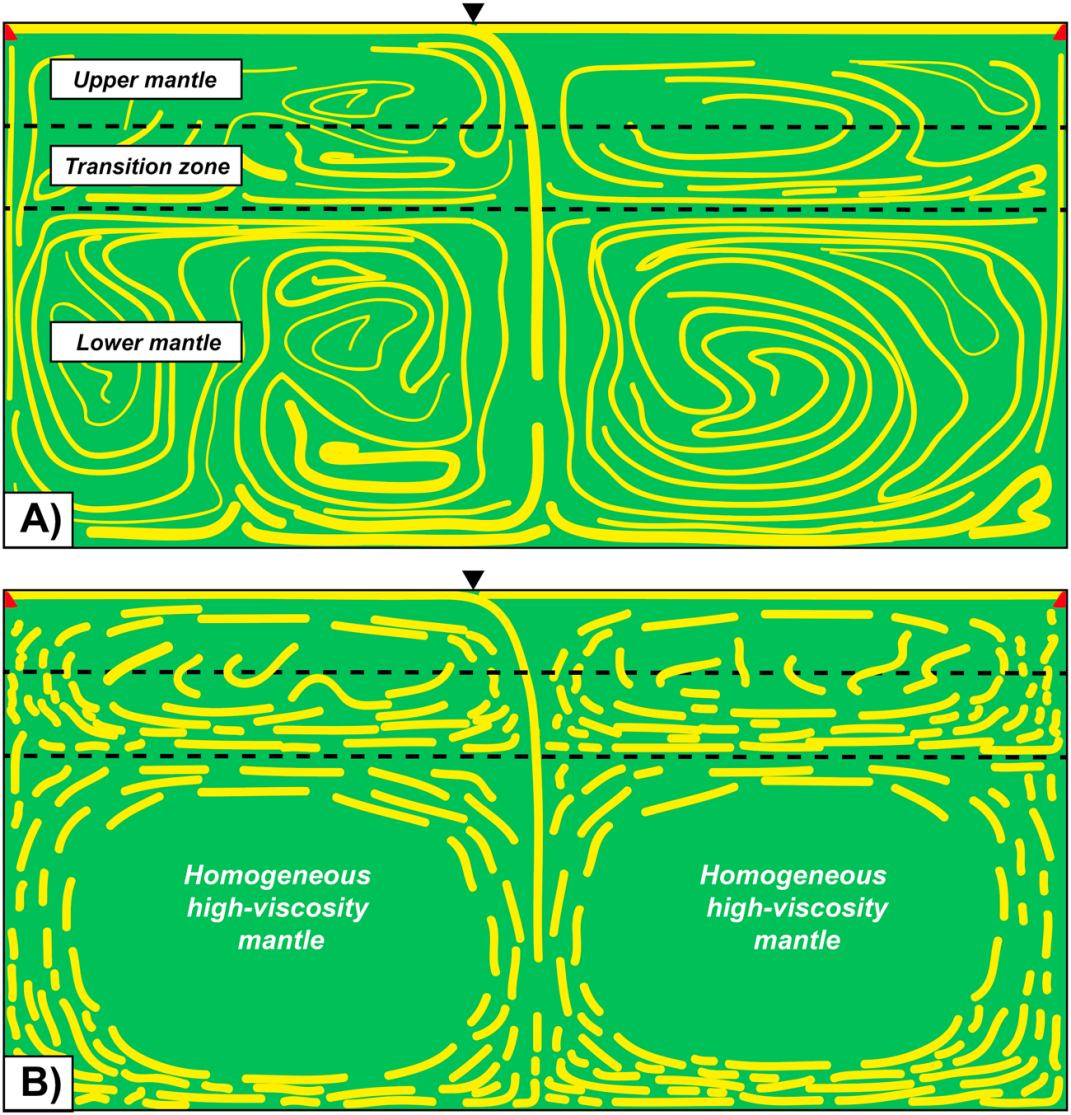
Schematic representation of compositional heterogeneities in the convective mantle. Basalt heterogeneities (yellow) tend to accumulate at the base of the transition zone and lower mantle due to density contrasts with the ultramafic rocks. (a) When viscosity is weakly dependent on compositional heterogeneities, basalt layering is laterally continuous. (b) Relatively high contrasts in viscosity among bridgmanite‐enriched domains, basalts, and surrounding depleted mantle regions prevent efficient lower mantle mixing and might cause necking/boudinage of basalt layers.

It is important to note that more recent numerical studies have demonstrated that relatively small changes in viscosity such as those associated with compositional heterogeneities in the lower mantle can prevent efficient mantle mixing (Ballmer et al., 2017). As a consequence, grain‐scale and rock‐scale layering may not be so widespread but present only within narrow conduits of upwelling and downwelling mantle material surrounding relatively undeformed lower mantle portions (the so‐called bridgmanite‐enriched ancient mantle structures; Ballmer et al., 2017) where recovery mechanisms may have erased any existing (intrinsic and extrinsic) fabric (Figure 8). This could be one of the reasons why the lower mantle is mostly seen as isotropic in seismological analyses.

Besides the dependency on contrasts in isotropic elastic moduli, strength of the SPO and shapes of the compositional heterogeneities, extrinsic anisotropy, when present, is also a strong function of the incidence angle. We have shown that little or no *S* wave anisotropy and *P* wave radial anisotropy are produced for 30–60° incidence angles (although *P* wave azimuthal anisotropy is still a large fraction of the maximum). Considering that most subducting slabs dip with a 30–60° angle, it follows that, even when the contrast in seismic velocities is large, the slab parallel quasi‐laminated structures would not affect significantly horizontally traveling surface waves. Similarly, SKS waves, which are mostly sensitive to the (receiver‐side) uppermost 500 km of the mantle (Sieminski et al., 2007) through which they travel subvertically, will be significantly affected only by subvertical layered structures. However, even when this is the case, the anisotropy generated by multicomponent systems analogous to those determined with wave scattering data (Furumura & Kennett, 2005; Sun et al., 2014) is quite small (0.1–0.2%, Figure 6). Hence, the trench‐parallel SKS anisotropy frequently measured in forearcs (Long & Silver, 2008) is probably the expression of the mantle LPO (Song & Kawakatsu, 2012) together with, perhaps, the serpentinized and fluid‐filled fractures present within and right above the dehydrating slab at intermediate‐depths (Faccenda et al., 2008; Healy et al., 2009). Considering that, for a given compositional heterogeneity, *P* wave extrinsic anisotropy is generally lower than *S* wave extrinsic anisotropy, *P* wave anisotropy should also reflect the rock intrinsic anisotropy together with contributions from the serpentinized and fluid‐filled fractures.

Our analysis also shows that extrinsic anisotropy can form also in a compositionally homogeneous mantle, where velocity variations associated with major phase transitions can generate up to 1% of positive radial anisotropy; this is consistent with previous studies that showed extrinsic anisotropy generated near seismic discontinuities (e.g., Wang et al., 2015).

## 5 Conclusions

We have quantified the potential amount of extrinsic anisotropy related to grain‐scale and rock‐scale mantle compositional heterogeneities. The magnitude of extrinsic anisotropy depends on the contrast in isotropic wave speeds among the different materials, their relative volume fractions, the shape of the heterogeneities, and wave incidence angle. Bearing in mind that it is unclear whether laterally continuous compositional layering is widespread through the mantle, we have shown that, when present, subsolidus rock‐scale SPO generally produces minor extrinsic anisotropy (<0.5–1%), so that it is hard to distinguish mantle compositional layering in most if not all the mantle. The presence of “exotic” compositional heterogeneities such as sediments and serpentinites can produce significant extrinsic anisotropy because of the large contrasts in elastic moduli with dry mafic and ultramafic rocks, but the effect would be localized within and right above the subducting slab. It follows that rock‐scale compositional heterogeneities can be determined mainly from studies of *P* and *S* wave scattering.

In contrast, grain‐scale SPO, which is the expression of the recent deformation history, is a mechanism that potentially can produce detectable extrinsic seismic anisotropy. Large contrast in viscosity among the different phases of the aggregate will prevent the formation of planar fabrics due to the presence of elongated ribbons of the hard (matrix or inclusion) phase. Foliated pyrolitic‐harzburgitic mantle or basalts could explain the strength of the radial anisotropy measured in the uppermost lower mantle in between the postspinel and postgarnet phase transitions. The *P* and *S* wave anisotropy measured in the intrinsically isotropic lower transition zone could be explained with grain‐scale SPO in MORB material accumulated above the 660‐km discontinuity. Thus, seismic anisotropy measured in these mantle regions could allow deciphering the strain‐induced grain‐scale SPO and hence the recent deformational history.

Alternatively, we have shown that up to 1% of positive radial anisotropy can be generated in a compositionally homogeneous mantle around the transition zone boundaries due to *S* wave finite‐frequency sensitivities and velocity variations associated with major phase transitions.

## Supporting information



Supporting Information S1
