# Supplementary material for: Extrinsic Elastic Anisotropy in a Compositionally Heterogeneous Earth's Mantle
Source: J Geophys Res Solid Earth. 2019 Feb 8;124(2):1671–87. doi: 10.1029/2018JB016482 (PMC6472509; doi:10.1029/2018JB016482)
Supplement: Supplementary file 1 — Supporting Information S1 [file JGRB-124-1671-s001.docx]

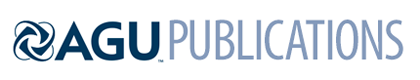


*[JGR – Solid Earth]*

Supporting Information for

**Extrinsic elastic anisotropy in a compositionally**

**heterogeneous Earth’s mantle**

Manuele Faccenda^1^, Ana MG Ferreira^2,3^, Nicola Tisato^4,5^, Carolina Lithgow-Bertelloni^2^, Lars Stixrude^2^, Giorgio Pennacchioni^1^

*^1^ Dipartimento di Geoscienze, Università di Padova, via Gradenigo 6, Padova, Italy*

*^2^ Department of Earth Sciences, University College London, London WC1E 68T, UK*

*^3^ CERIS, Instituto Superior Tecnico, Universidade de Lisboa, Av. Rovisco Pais 1, 1049-001 Lisboa, Portugal*

*^4^ Department of Geological Sciences, Jackson School of Geosciences, University of Texas, Austin, USA*

*^5^ University of Toronto, Department of Civil Eng., Toronto, Canada*

**Contents of this file**

Text S1 to S5

Figures S1 to S4

**Additional Supporting Information (Files uploaded separately)**

Captions for Movies S1 to S2

**Introduction**

In the following sections we provide details about the 3D mechanical modelling used to simulate foliated-lineated fabrics, the seismic anisotropy parameters quantifying extrinsic anisotropy, the effective medium theories and the weighting functions used to calculate the elastic properties of layered and non-layered media, and the seismic wave propagation modelling performed with Sofi-3D

**Text S1. Modelling grain-scale fabrics**

The development of grain-scale fabrics was simulated with 3D mechanical modelling of Newtonian viscous deformation in two-phase aggregates. Simulations were run by using I3MG (Gerya, 2010) modified to account for periodic boundary conditions. The initial setup is displayed in Fig. S1, while the fabrics after $\gamma$ = 10 in Fig. 2 ($\gamma$ is twice the bulk shear strain). The adimensional numerical domain is cubic (length L = 1 discretized with 245 nodes) and periodic along the X direction. Simple shear deformation is generated by moving two thin (thickness of 0.01) and stiff ($\eta_{p}=10000$) plates in opposite directions at a rate of $V_{x}=\pm$ 1, yielding a bulk shear strain rate $\dot{\varepsilon}_{xz\_bulk}$ = 1.02 (Fig. S1). In between the sliding plates, the two-phase aggregate is made of initially spherical, randomly distributed and isolated inclusions (volume fraction $\phi_{i}= 30\%$, radius in the 0.1-0.3 range) surrounded by a more abundant matrix ($\phi_{m}= 70\%$).

*
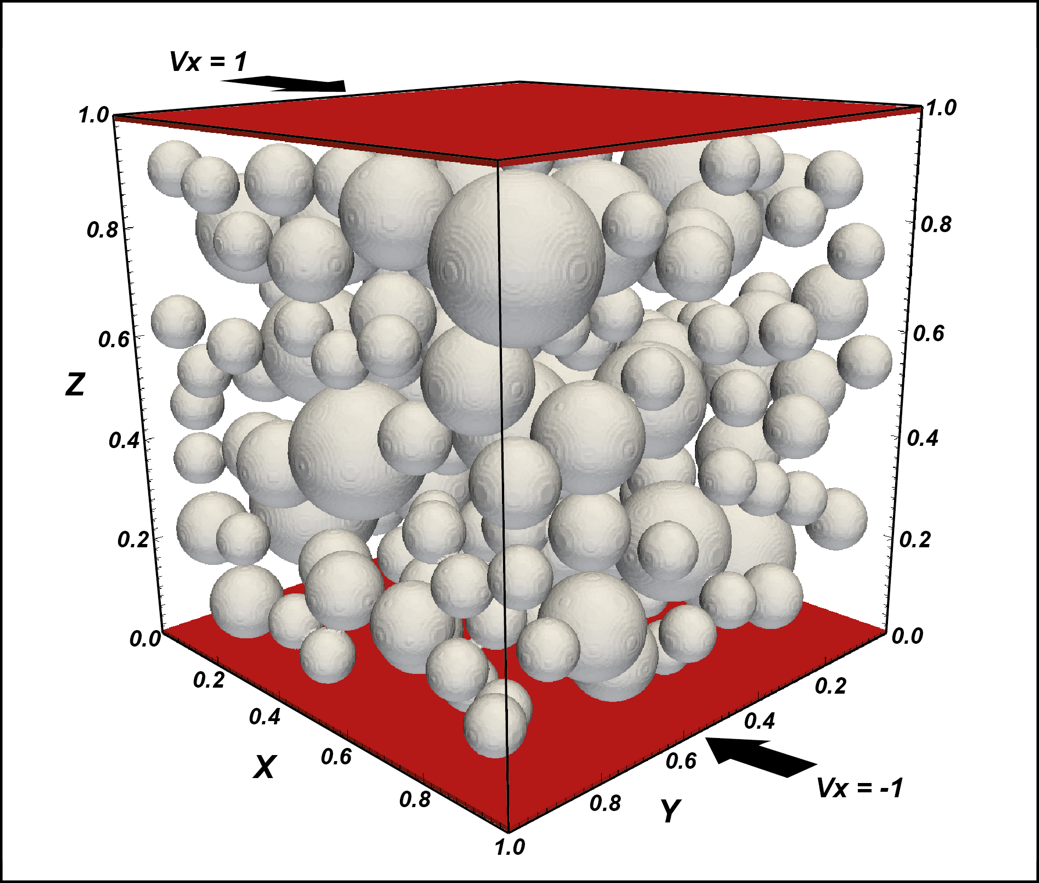

Fig. S1 – Initial setup for the 3D mechanical modelling of grain-scale fabric development.*

**Text S2. Definition of seismic anisotropy quantities**

The elastic behaviour of a transversely isotropic medium with a vertical symmetry axis (VTI) is defined by the Love elastic constants *A*, *C*, *F*, *L*, *N*. In Voigt notation the elastic tensor of the VTI medium is:

$$\begin{aligned} \begin{aligned} C_{ij}=\left| \begin{matrix} C_{11} & C_{12} & C_{13} & 0 & 0 & 0 \\ C_{21} & C_{22} & C_{23} & 0 & 0 & 0 \\ C_{31} & C_{32} & C_{33} & 0 & 0 & 0 \\ 0 & 0 & 0 & C_{44} & 0 & 0 \\ 0 & 0 & 0 & 0 & C_{55} & 0 \\ 0 & 0 & 0 & 0 & 0 & C_{66} \end{matrix} \right|=\left| \begin{matrix} A & A-2N & F & 0 & 0 & 0 \\ A-2N & A & F & 0 & 0 & 0 \\ F & F & C & 0 & 0 & 0 \\ 0 & 0 & 0 & L & 0 & 0 \\ 0 & 0 & 0 & 0 & L & 0 \\ 0 & 0 & 0 & 0 & 0 & N \end{matrix} \right|\# \end{aligned}\#S2.1 \end{aligned}$$

For any elastic tensor $C_{ij}$ defined in Voigt notation, the azimuthally averaged Love elastic constants are (Montagner and Nataf, 1986):

$$A=\frac{3}{8}\left( C_{11}+C_{22} \right)+\frac{1}{4}C_{12}+\frac{1}{2}C_{66}$$

$$C=C_{33}$$

$$\begin{aligned} F=\frac{1}{2}\left( C_{13}+C_{23} \right)\#S2.2 \end{aligned}$$

$$L=\frac{1}{2}\left( C_{44}+C_{55} \right)$$

$$N=\frac{1}{8}\left( C_{11}+C_{22} \right)-\frac{1}{4}C_{12}+\frac{1}{2}C_{66}$$

**Text S3. Effective Medium Theories**

*S3.1 Smoothed Transversely Isotropic Long Wavelength Equivalent (STILWE)*

Introduced by (*Backus, 1962*), the STILWE is widely used to estimate the elastic properties of a stack of isotropic layers whose thickness is much smaller than the seismic wavelength. The equivalent elastic moduli of the homogeneous transversely isotropic medium are algebraic combinations of algebraic combinations of two elastic parameters ($\lambda$, $\mu$ or $\theta={Vs}^{2}/{Vp}^{2}$). For a VTI medium, the elastic constants can be computed by using averages $\left\langle\cdot\right\rangle$ of $\mu$ and $\theta$ as (Backus, 1962):

$$A=4\left( N-S \right)+R^{-1}\left( 1-2T \right)^{2}$$

$$C=R^{-1}$$

$$\begin{aligned} F=R^{-1}\left( 1-2T \right)\#S3.1 \end{aligned}$$

$$L=\left\langle\frac{1}{\mu} \right\rangle^{-1}$$

$$N=\left\langle\mu\right\rangle$$

where:

$$R=\left\langle\theta/\mu\right\rangle$$

$$\begin{aligned} S=\left\langle\theta\mu\right\rangle\#S3.2 \end{aligned}$$

$$T=\left\langle\theta\right\rangle$$

The range of $\mu$ and $\theta$ for which the STILWE is stable is $0\leq\theta\leq3/4$ and $0\leq\mu<+\infty$.

In case of a Periodic Isotropic Two-Layered (PITL) medium, we can define the relative volume fraction of medium 1 and 2 as $x_{1}$ and $x_{2}=1-x_{1}$. It can be shown analytically that for any contrast in isotropic elastic moduli both $R_{P}$ and $R_{S}$ vary parabolically with the relative volume fraction, such that they are null when $x_{1}=0$ or $x_{2}=0$, and maximum when $x_{1}=x_{2}=0.5$ (Fig. S2).


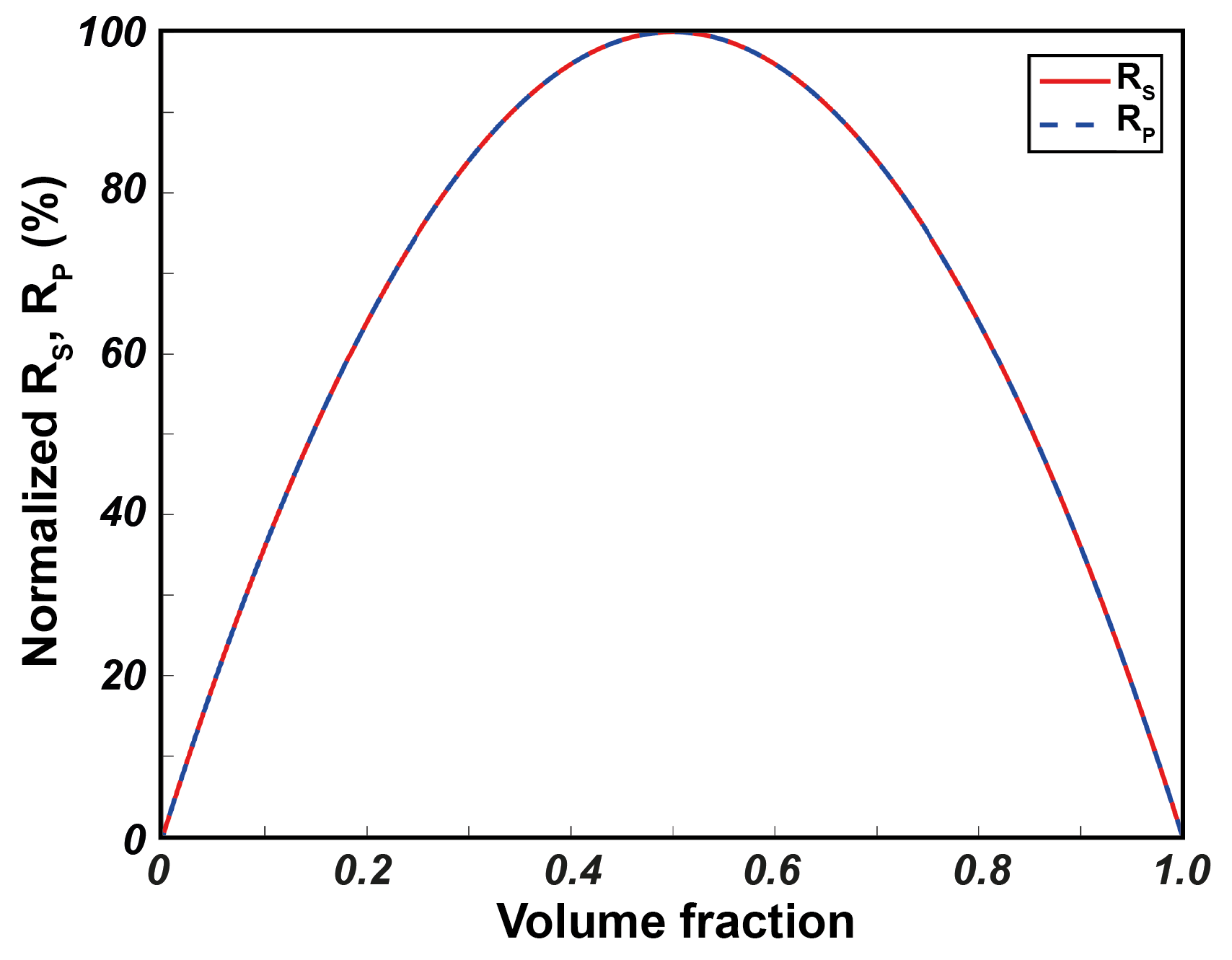


*Figure S2 - R_P_ and R_S_ normalized by their maximum value as a function of volume fraction of either medium 1 or 2.*

*S3.2 Differential Effective Medium (DEM) Theory*

The tensorial formulation for DEM is (McLaughlin, 1977):

$$\begin{aligned} \frac{d\boldsymbol{C}^{\boldsymbol{DEM}}}{dV}=\frac{1}{\left( 1-V \right)}\left( \boldsymbol{C}^{\boldsymbol{i}}-\boldsymbol{C}^{\boldsymbol{DEM}} \right)\boldsymbol{A}^{\boldsymbol{i}}\#S3.2.1a \end{aligned}$$

$\begin{aligned} \boldsymbol{A}^{\boldsymbol{i}}=\left[ \boldsymbol{I}+\boldsymbol{G}^{\boldsymbol{s}}\left( \boldsymbol{C}^{\boldsymbol{i}}-\boldsymbol{C}^{\boldsymbol{DEM}} \right) \right]^{-1}\boldsymbol{\#}S3.2.1b \end{aligned}$

where $\boldsymbol{C}^{\boldsymbol{i}}$ and $\boldsymbol{C}^{\boldsymbol{DEM}}$ are the 4^th^-order elastic tensors of the inclusion and of the effective medium, respectively, $V$ is the volume fraction of the inclusion, $\boldsymbol{A}^{\boldsymbol{i}}$ is the ratio of strain inside the inclusion to the strain in the host medium, $\boldsymbol{I}$ is the symmetric fourth-rank unit tensor, $\boldsymbol{G}^{\boldsymbol{s}}$ is the symmetric Green’s interaction tensor (Hornby et al., 1994; Mainprice, 2007):

$$\begin{aligned} G_{ijkl}^{s}=\frac{1}{2}\left( G_{ikjl}+G_{jkil} \right)\#S3.2.2a \end{aligned}$$

$$\begin{aligned} G_{ijkl}=\frac{1}{4\pi}\int_{0}^{\pi} sin\theta d\theta\int_{0}^{2\pi} \left( {K_{ij}^{-1}\left( x \right)x}_{k}x_{l} \right)d\phi\#S3.2.2b\# \end{aligned}$$

Here, the Green’s interaction tensor $\boldsymbol{G}$ depends on the inclusion geometry and matrix elastic tensor through the Christoffel stiffness tensor $K_{ik}\left( x \right)=C_{ijkl}^{DEM}x_{j}x_{l}$ and directions $x_{1}=sin\theta cos\phi/a_{1}$, $x_{2}=sin\theta sin\phi/a_{2}$and $x_{3}=cos\theta/a_{3}$, where $a_{1}, a_{2}, a_{3}$ are the semiaxes of the ellipsoidal inclusion.

Eq. S3.2.1a is solved with a 1^st^–order in time, 4^th^-order in space Runge-Kutta method by setting the matrix as the initial effective medium and then by progressively increasing the volume fraction of the inclusion. It is important to note that, for any inclusion concentration, the host medium is always fully interconnected while the inclusions remain isolated.

**Text S4. Weighting functions**

In order to estimate the polarization anisotropy along the reference pyrolytic mantle profile displaying a perfectly layered grain‐scale SPO (see Fig. 5), we use two different sets of weighting functions: (i) Gaussian functions with half-width of 15 km and of 50 km; and, (ii) Solutions of the radial wave equation, following (Capdeville et al., 2013). The latter weighting functions are defined as:

$$\begin{aligned} w_{n1,n2,r0}\left( r \right)=\sum_{n} u_{n}\left( r \right)w_{n1,n2}\left( n \right)d_{r0,n}\#S4.1 \end{aligned}$$

where

$$\begin{aligned} d_{r0,n}=\int_{R} \delta\left( r-r_{0} \right)u_{n}\left( r \right)dr\#S4.2 \end{aligned}$$

$$\begin{aligned} w_{n1,n2}\left( n \right)={\frac{1}{\sqrt{2\pi\sigma^{2}}}e}^{-\frac{1}{2}\left( \frac{n}{\sigma} \right)^{2}}\#S4.3 \end{aligned}$$

$$\begin{aligned} u_{n}\left( r \right)=rU_{0}\left( r,{{}_{n}\omega}_{0} \right)\#S4.4 \end{aligned}$$

$U_{0}\left( r,{{}_{n}\omega}_{0} \right)$ is the radial eigenfunction satisfying the wave equation in a spherically symmetric Earth model, ${{}_{n}\omega}_{0}$ is the corresponding eigenfrequency for radial order *n* and for angular order *l=*0. We use $\sigma=50$, which leads to weighting functions with width that increases as a function of depth. This reflects the decreasing spatial resolution of seismic waves as depth increases (e.g., Ferreira et al., 2010). Since they satisfy the wave equation, the weighting functions based on the radial normal modes are more realistic than using Gaussian functions with constant half-width. In particular, as shown by (Capdeville et al., 2013), these weighting functions get wider as depth increases (Figure S3), and thus naturally account for a change in minimum wavelength as a function of depth.

*Figure S3. Two examples of weighting functions built using radial eigenfunctions (Eq. S4.1) for two depth values: 500 km (black line), 1500 km (red line) using a normal mode basis computed for the pyrolitic mantle model considered in the main text. As depth increases, the weighting function is wider.*

**Text S5. Modelling seismic wave propagation**

We simulated wave propagation in the synthetic media by means of Sofi3D (Bohlen, 2002). Sofi3D solves wave propagation in a three-dimensional space using a time-explicit finite difference method. To limit numerical dispersion, we used an eight- and second-order approximation in space and time, respectively. Samples were 236x236x236 voxels and each voxel was 1x1x1 mm in size. We assigned $K=120$ GPa and $\mu=78$ GPa to the inclusion particles and $K=80$ GPa and $\mu=42$ GPa to the matrix particles of the 3D mechanical models, and interpolate them to the Sofi3D numerical grid. In addition we created a reference layered model with 28% volume fraction of the fast phase (Fig. S3). This large contrast in isotropic elastic moduli is needed to observe seismic anisotropy with the used model dimension and time step.

Synthetic sources and receivers were placed on opposite sides of the sample and placed on a grid of 22x22 nodes. Nodes were 10 mm apart from each other. For Vs and Vp tests sources generated a shear and compressive displacement, respectively. The source wavelet was a sin^3^ with centre frequency of 50 kHz. Such a frequency created a wavelength that was shorter than the sample but longer than the heterogeneity thicknesses. Thus, we avoided near-field effects and heterogeneities behaved as effective media. To limit lateral reflections and multiples, external faces were added with paddings having width of 32 nodes, and we assigned absorbing properties to the paddings (DAMPING=8; see the [Sofi3D manual](https://git.scc.kit.edu/GPIAG-Software/SOFI3D/tree/overnightbuilt/doc/guide_sofi3D)). We simulated wave propagation for 40 µs and final waveforms were generated by averaging the 484 particle velocity signals recorded by the receivers. Time step was 0.01 µs and first arrival picking was determined by thresholding the waveforms. Threshold was equal for all the tests and applied to amplitude-normalized wavelets. Finally, anisotropy of shear waves (AVs) was estimated comparing shear waves that were propagated in the same direction but had different polarizations using eq. 2.

The velocity of the fast or the slow shear wave was calculated as:

$$\begin{aligned} {Vs}_{1\bigvee2}=\frac{L}{t_{1\bigvee2}}\#S5.1 \end{aligned}$$

Where L is the sample length (236 mm) and $t_{1\bigvee2}$ is the time arrival of the fast or the slow wave (i.e., t_1_ or t_2_). There was no uncertainty on the sample length, while we established that a reasonable variation of the picking threshold would have caused an uncertainty on the absolute shear wave arrival time $(\delta t)$ of ±0.25 µs. Uncertainty for shear wave velocities was then estimated as:

$$\begin{aligned} \delta{Vs}_{1\bigvee2}={Vs}_{1\bigvee2} \frac{\delta t}{t_{1\bigvee2}} \#S5.2 \end{aligned}$$

Furthermore, we assumed no uncertainty on the difference between time arrivals (i.e., no uncertainty on $\Delta t=t_{1}-t_{2}$). This assumption implies that the same error is made in picking the wave arrivals of fast and slow shear waves. The assumption is justified as fast and slow shear waves have very similar wavelets and amplitudes, and we use the same threshold for both waves, thus a variation in the picking threshold would cause a similar variation in both the arrival times. As a consequence, since:

$$\begin{aligned} \frac{1}{{Vs}_{1}}-\frac{1}{{Vs}_{2}}=\frac{t_{1}}{L}-\frac{t_{2}}{L}\to{Vs}_{1}-{Vs}_{2}={Vs}_{1}{Vs}_{2}\left( \frac{t_{2}-t_{1}}{L} \right)=\Delta V_{s} \#S5.3 \end{aligned}$$

and the error on ${Vs}_{1}{Vs}_{2}$ is:

$\delta{Vs}_{1}{Vs}_{2}={Vs}_{1}{Vs}_{2}\sqrt{\left( \frac{\delta{Vs}_{1}}{{Vs}_{1}} \right)^{2}+\left( \frac{\delta{Vs}_{2}}{{Vs}_{2}} \right)^{2}}$,

the error on $\Delta V_{s}$ is:

$$\begin{aligned} \delta\Delta V_{s}=\Delta V_{s}\frac{\delta{Vs}_{1}{Vs}_{2}}{{Vs}_{1}{Vs}_{2}}=\Delta V_{s}\sqrt{\left( \frac{\delta{Vs}_{1}}{{Vs}_{1}} \right)^{2}+\left( \frac{\delta{Vs}_{2}}{{Vs}_{2}} \right)^{2}} \#S5.4 \end{aligned}$$

and finally the uncertainty on AVs is:

$$\begin{aligned} \delta AVs=AVs\sqrt{\left( \frac{\delta\Delta V_{s}}{\Delta V_{s}} \right)^{2}+\left( \frac{\delta{Vs}_{2}}{{Vs}_{2}} \right)^{2}} \#S5.5 \end{aligned}$$

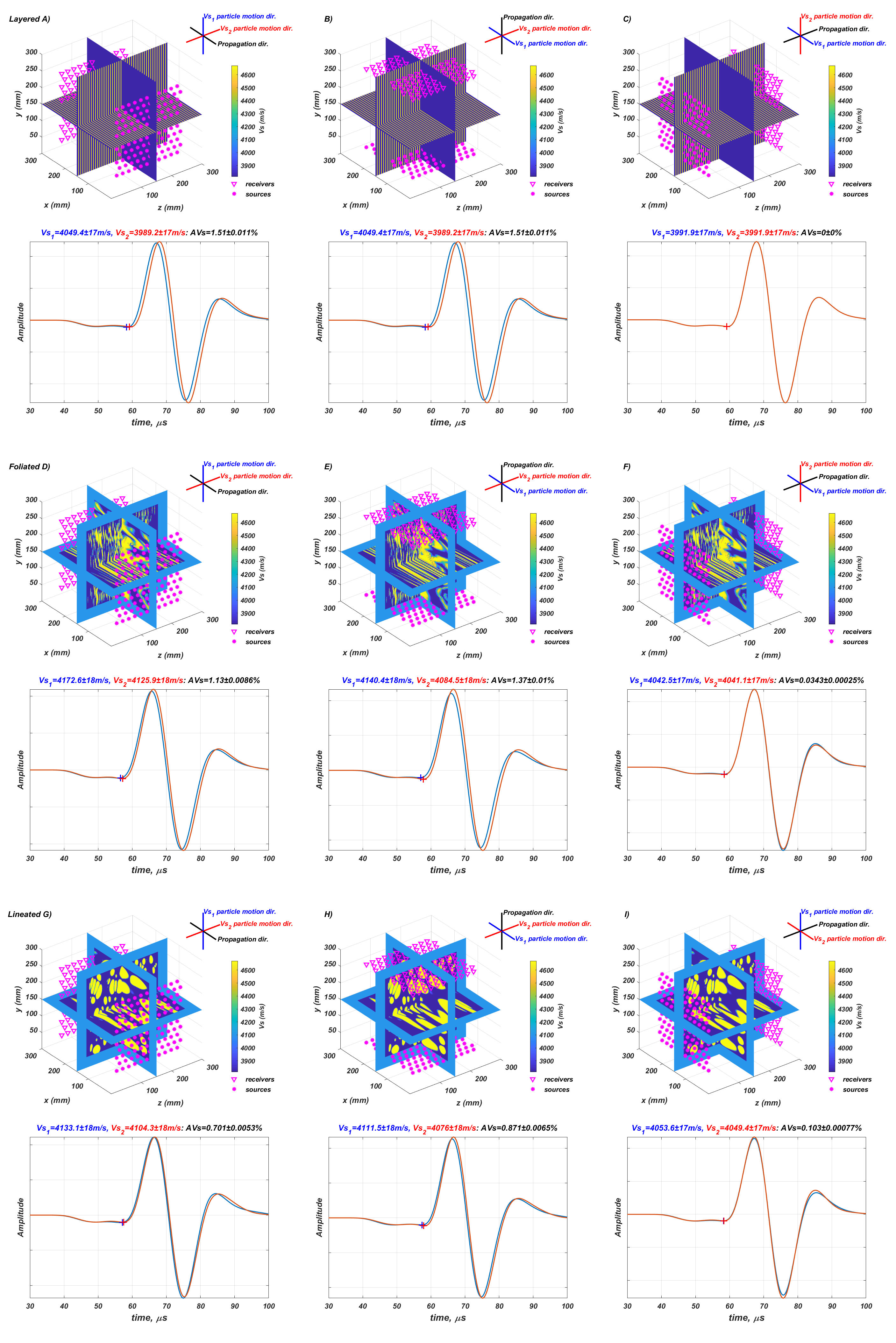


*Figure S4 – Summary of the seismic wave propagation modelling results. Seismic wavefields propagate along the X (1^st^ column), Y (2^nd^ column) and Z (3^rd^ column) directions through a layered (1^st^ row), foliated (2^nd^ row) and lineated (3^rd^ row) medium.*

**References**

Backus, G.E., 1962. Long-wave elastic anisotropy produced by horizontal layering. J. Geophys. Res. 67, 4427–4440.

Bohlen, T., 2002. Parallel 3-D viscoelastic finite difference seismic modelling. Comput. Geosci. 28, 887–899. https://doi.org/10.1016/S0098-3004(02)00006-7

Capdeville, Y., Stutzmann, E., Wang, N., Montagner, J.-P., 2013. Residual homogenization for seismic forward and inverse problems in layered media. Geophys. J. Int. 194, 470–487.

Ferreira, A.M.G., Woodhouse, J.H., Visser, K., Trampert, J., 2010. On the robustness of global radially anisotropic surface wave tomography. J. Geophys. Res. 115, 1–16.

Gerya, T. V, 2010. Introduction to numerical geodynamical modelling. Cambridge University Press.

Hornby, E.B., Schwartz, L.M., Hudson, J.A., 1994. Anisotropic effective-medium modeling of the elastic properties of shales. Geophysics 59, 1570–1583.

Mainprice, D., 2007. Seismic anisotropy of the deep Earth from a mineral and rock physics perspective. Treatise Geophys. 2, 437–492.

McLaughlin, R.A., 1977. A study of the differential scheme for composite materials. Int. J. Eng. Sci. 15, 237–244.

Montagner, J.P., Nataf, H.C., 1986. A simple method for inverting the azimuthal anisotropy of surface waves. J. Geophys. Res 91. https://doi.org/doi:10.1029/JB091iB01p00511

Movie S1. Development of foliated fabric when inclusions are ten times weaker than the matrix. Initial setup is shown in Fig. S1.

Movie S2. Development of lineated fabric when inclusions are ten times stronger than the matrix. Initial setup is shown in Fig. S1.
